# Antimicrobial Compounds Effective against *Candidatus* Liberibacter asiaticus Discovered via Graft-based Assay in Citrus

**DOI:** 10.1038/s41598-018-35461-w

**Published:** 2018-11-23

**Authors:** Chuanyu Yang, Yun Zhong, Charles A. Powell, Melissa S. Doud, Yongping Duan, Youzong Huang, Muqing Zhang

**Affiliations:** 1 State Key Laboratory for Conservation and Utilization of Subtropical Agro-Biological Resources, Guangxi University, Nanning, Guangxi 530005 China; 2 Indian River Research and Education Center-Institute of Food and Agricultural Sciences, University of Florida, 2199 South Rock Rd, Fort Pierce, FL 34945 USA; 3 US Department of Agriculture-Agricultural Research Service-US Horticultural Research Laboratory, 2001 South Rock Rd, Fort Pierce, FL 34945 USA; 4 Fruit research institute, Fujian Academy of Agricultural Sciences, Fuzhou, Fujian, 350003 China; 5 Institute of Fruit Tree Research, Guangdong Academy of Agricultural Sciences, Guangzhou, 510640 Guangdong China

## Abstract

Huanglongbing (HLB), the most destructive citrus disease, is caused by three species of phloem-limited *Candidatus* Liberibacter. Chemical control is a critical short-term strategy against *Candidatus* Liberibacter asiaticus (Las). Currently, application of antibiotics in agricultural practices is limited due to public concerns regarding emergence of antibiotic-resistant bacteria and potential side effects in humans. The present study screened 39 antimicrobials (non-antibiotics) for effectiveness against Las using an optimized graft-based screening system. Results of principal component, hierarchical clustering and membership function analyses demonstrated that 39 antimicrobials were clustered into three groups: “effective” (Group I), “partly effective” (Group II), and “ineffective” (Group III). Despite different modes of action, 8 antimicrobials (aluminum hydroxide, D,L-buthionine sulfoximine, nicotine, surfactin from *Bacillus subtilis*, SilverDYNE, colloidal silver, EBI-601, and EBI-602), were all as highly effective at eliminating or suppressing Las, showing both the lowest Las infection rates and titers in treated scions and inoculated rootstock. The ineffective group, which included 21 antimicrobials, did not eliminate or suppress Las, resulting in plants with increased titers of *Candidatus* Liberibacter. The other 10 antimicrobials partly eliminated/suppressed Las in treated and graft-inoculated plants. These effective antimicrobials are potential candidates for HLB control either via rescuing infected citrus germplasms or restricted field application.

## Introduction

Citrus Huanglongbing (HLB) is an serious citrus disease and has caused enormous economic losses to citrus industry in the world^1,2^. Citrus HLB has been in China for at least 100 years^3^. In Florida,USA, since HLB was first discovered in August 2005, citrus acreage and production in Florida have declined from 750,000 acres and 170 million boxes to 520,000 acres and less than 80 million boxes in 2015–2016, respectively^4^. And the Florida citrus industry has lost over 50% of its citrus plants, and production is decreasing at an alarming rate^5^.

HLB is caused by three species of uncultured, phloem-restricted proteobacteria in the *Candidatus* Liberibacter genus, *L. asiaticus* (Las), *L. americanus*, and *L. africanus*^1,6,7^, and is transmitted by either *Diaphorina citri* or *Trioza erytreae*^8^. Effective strategies against Las bacterium in citrus production are still limited, and breeding resistant citrus varieties is considered to be the most efficient and sustainable strategy against HLB. Thus, traditional citrus breeding has often been limited, due to polyembryony, pollen-ovule sterility, sexual and graft incompatibilities, and extended juvenility^9^. To date, there are still no commercial genetically modified citrus varieties available due to lack of consumer acceptance of genetically modified organisms. Therefore, it will likely take many years to release an HLB-resistant citrus cultivar.

Chemical control is considered to be an effective short-term strategy for combating citrus HLB. In our previous studies, a graft-based chemical control method was developed and applied for screening novel effective antibiotics against HLB^10,11^. These antibiotics (ampicillin, carbenicillin, penicillin, cephalexin, rifampicin, and sulfadimethoxine) have been confirmed to be effective against Las bacterium^11^. The results of oil-in-water and water-in-oil nanoemulsion delivery of the effective antibiotics into citrus phloem from bark and foliar, respectively, indicated that these nanoemulsions enhanced the therapeutic efficacy of the antibiotics against Las bacterium^12,13^. In addition, several studies also demostrated application of antibiotics and plant defense inducers by trunk-injection also suppress Las titer in HLB-affected citrus in the field^14–16^.

Currently, application of antibiotics in agricultural practices has become limited due to public concerns regarding the emergence of antibiotic-resistant bacteria and potential side effects in humans. In recent, under the emergency Exemption provisions of Federal Insecticide, Fungicide, and Rodenticide Act (FIFRA), Florida has declared an HLB crisis that allows use of the antibiotics including: streptomycin sulfate (Fire Wal 50WP. AgroSource, Inc.), oxytetracycline hydrochloride (FireLine 17WP, AgroSource, Inc.), and oxytetracycline calcium complex (Mycoshield, Nufarm Americas, Inc.) for controlling citrus HLB by foliar application in Florida. In the previous studies, streptomycin sulfate and oxytetracycline can suppress Las titer in greenhouse and field^17–20^, thus, tetracycline and streptomycin were only bacteriostatic rather than bactericidal^11,19^. It is necessary for continuous application of these two antibiotics to suppress the disease, thus, frequent applications are high cost and may result in the emergence of antibiotic-resistant bacteria. Although the antibiotics screened in our previous study have been shown to be effective against Las and less phytotoxic^11^, their application to citrus crops in commercial groves has not yet been approved by the US Environment Protection Agency or other regulatory agencies. As with any new active ingredient, registration of these active ingredients would take many years. Considering the long approval period, potential health risks, and lack of evidence regarding their superiority to other chemicals currently used in plant agriculture, use of these antibiotics is not viable/practical for HLB. Nowadays, HLB is seriously threating citrus industry in Florida and other regions of the world. Therefore, screening of non-antibiotic or other chemical compounds that have already been registered for fruit tree production and can reduce the emergence on antibiotic-resistant bacteria is urgently needed for the survival of the Florida citrus industry. In the present study, 39 antimicrobial (nonantibiotic) compounds (including natural product, antimicrobial metals, and commercial product), which can reduce risk of emergence of antibiotic resistant bacteria and potential side effects in humans, were evaluated for effectiveness against HLB and phytotoxicity via an optimized graft-based assay.

## Materials and Methods

### Antimicrobial compounds and working concentrations

Antimicrobial compounds and their concentrations used for screening were selected according to suggestions from the InnoCentive group who have cooperated with the Citrus Research and Development Foundation in Florida (USA). A call was solicited worldwide for suggestions of antimicrobial compounds that may combat Las bacterium infection. Based on the suggestions of range of the concentration received, the citrus scion (rough lemon, *Citrus limonum*) were soaked into antimicrobial compounds solution at different concentration for 24 hours. Then, based on observation of phytotoxicity (such as leaf wilting) on citrus scion, the concentration of antimicrobial compounds would be determined for optimized graft-based assay. In this study, antibacterial activity of 39 antimicrobial compounds were evaluated by optimized graft-based assay. Important information pertaining to each compound is provided in Table 1.

**Table 1:** Information of chemical compounds screened for control of citrus Huanglongbing.

Code	Chemcial name	Company	Work conc.	Solvent	Type	Mode of action
AL	Aluminum hydroxide	Sigma Aldrich	200 mg/l	water	metal	distruption of membrane structure^38^
Amp	Ampicillin	Fisher Scientific	1000 mg/l	water	positive control	distruption of membrane structure^63^
AZA	Azadirachtin	Sigma Aldrich	100 mg/l	ethanol	natural product	distruption of membrane structure^64^
CARV	Carvacrol	Sigma Aldrich	100 mg/l	water	natural product	distruption of membrane structure^58^
MESO	Meso-erythritol	Fisher Scientific	3000 mg/l	water	natural product	distruption of membrane structure^65^
PCY	P-cymene	Santa Cruz Biotechnology	100 mg/l	water	natural product	distruption of membrane structure^58^
PDL	Poly-D-lysine	Sigma Aldrich	100 mg/l	water	natural product	distruption of membrane structure^66^
PLA	Poly-l-arginine	Sigma Aldrich	100 mg/l	water	natural product	distruption of membrane structure^66^
SFC	Surfactin from *bacillus subtitlis*	Sigma Aldrich	10 mg/l	ethanol	natural product	distruption of membrane structure^51^
THU	Thujone	Sigma Aldrich	100 mg/l	ethanol	natural product	distruption of membrane structure^67^
NS	nanosilver	Attostat	5 mg/l	water	metal	distruption of membrane structure and energy metabolism^26–29^
SC	Silver collidal	Fisher Scientific	50 mg/l	water	metal	distruption of membrane structure and energy metabolism^26–29^
SD	SilverDyne	Word health alliance,international inc.	2 ml/l	water	metal	distruption of membrane structure and energy metabolism^26–29^
SDN	Silver,nanaparticle	Fisher Scientific	50 mg/l	water	metal	distruption of membrane structure and energy metabolism^26–29^
SN	Silver nitrate	Fisher Scientific	50 mg/l	water	metal	distruption of membrane structure and energy metabolism^26–29^
ABC	DL-2-aminobutyric acid	Sigma Aldrich	100 mg/l	water	natural product	induction of pathogenesis protein^68^
SAR	Proprietary SAR Inducer 2018A	Bayer CropScience	0.75 ml/l	water	commercial product	induction of pathogenesis protein^68^
BSO	DL-buthionine-sulfoximine	Sigma Aldrich	100 mg/l	DMSO	natural product	interference of activated oxygen metabolism^46–49^
BER	Berberine chloride	Sigma Aldrich	8 mg/l	ethanol	natural product	interference of energy metabolism^69^
MET	2-methyl-4-isothiazolin-3-one	Sigma Aldrich	4 ml/l	water	natural product	interference of energy metabolism^70^
NIC	Nicotine	Fisher Scientific	100 mg/l	ethanol	natural product	interference of energy metabolism^43–45^
EBI-601	EBI-601	Echelon Biosciences,Inc.	200 mg/l	water	commercial product	interference of nucleic acid metabolism^71^
EBI-602	EBI-602	Echelon Biosciences,Inc.	200 mg/l	water	commercial product	interference of nucleic acid metabolism^71^
2AC	2-amino-5-chlorobenzoxazole	Acros Organics	100 mg/l	DMSO	natural product	interference of nutrition metabolism^72^
HYD	Hydroxyurea crystalline	Fisher Scientific	500 mg/l	water	natural product	interference of nutrition metabolism^73^
INH	Isonicotinic acid hydrazide aminosalicylate	Fisher Scientific	100 mg/l	water	natural product	interference of nutrition metabolism^74^
QUI	Gossypol	Sigma Aldrich	100 mg/l	ethanol	natural product	interference of nutrition metabolism^75^
CRE	M-cresol	Fisher Scientific	4 ml/L	water	natural product	interference of other metabolism^76^
FA	Formic acid	Sigma Aldrich	1 ml/L	water	natural product	interference of other metabolism^77^
ZINEB	Zineb	Sigma Aldrich	250 mg/l	DMSO	commercial product	interference of other metabolism^78^
80WG	80WG	Bayer Crop Science	500 mg/l	water	commercial product	interference with cell wall synthesis^79^
BITC	Benzyl isothiocyanate	Sigma Aldrich	50 mg/l	water	natural product	interference with cell wall synthesis^80^
QUAD	Quadrix (cyproconzole)	Sigma Aldrich	1200 mg/l	ethanol	natural product	interference with cell wall synthesis^81^
SAP	Saponin	Santa Cruz Biotechnology	1000 mg/l	water	natural product	interference with cell wall synthesis^82^
20WP	20WP	Stamer 20 WP in Japan	2000mg/l	water	commercial product	unknown
EcoClean	EcoClean	EcoUSA	50 ml/l	water	commercial product	unknown
FT33	FT33-3	OCION	2000mg/l	water	commercial product	unknown
MA	MA-3	MagnaBon	1000 mg/l	DMSO	commercial product	unknown
Proud	Proud	BioHumaMetrics	10 ml/l	water	commercial product	unknown
PT81	PT81-3	OCION	1000 mg/l	water	commercial product	unknown
DMSO	DMSO	Fisher Scientific	1 ml/l	water	negative control	
CK-1	Water	—	—	—	negative control	
CK-2	Ethanol	Fisher Scientific	1 ml/l	water	negative control	

### Graft-based assay

Antibacterial activities of the compounds against Las and their phytotoxicity to citrus were evaluated by graft-based assay, according to our previous reports, with minor revisions^10,11^. Briefly, HLB-infected budsticks were collected from symptomatic rough lemon trees (*Citrus limonum*, “lemon #76”) at the US Department of Agriculture-Agricultural Research Service-US Horticultural Research Laboratory farm in Fort Pierce, FL (USA), and confirmed positive for Las by real-time quantitative polymerase chain reaction [qPCR]^10,21^. The budsticks were soaked in one of the chemical treatments listed in Table 1 (30 scions/treatment/concentration and two scion grafted into each rootstock) overnight in a fume hood under ventilation and continuous fluorescent light. Each soaked budstick was cut into a 2-bud scion and grafted onto individual 2-year-old HLB-free grapefruit (*Citrus paradisi* var. Duncan) rootstock seedlings. Then, grafts were covered with plastic tape for 21 days. To improve scion growth, new flush from the rootstocks was pruned immediately after grafting. Grafted plants were kept at 25 ± 2 °C under shade in an insect-proof greenhouse.

### Evaluation of chemical antibacterial activity and tree health

The antibacterial activities of chemicals tested against Las bacterium was determined by measuring the Las titer in both the grafted scion and rootstock via qPCR, according to Zhang’s protocol with minor modifications^10,11^. Briefly, five leaves were collected from both scion (rough lemon) and rootstock (grapefruit) at 6 months after grafting. Each leaf was rinsed three times with sterile water. Midribs were separated from the leaf samples and cut into 1.0 to 2.0 mm pieces. DNA was extracted from 0.1 g (fresh weight) of leaf midrib tissue using a DNeasy Plant Mini Kit (Qiagen, Valencia, CA) according to the manufacturer’s protocol. qPCR was performed with primers and probes (HLBas, HLBr and HLBp)^21^ for *Ca*. L. asiaticus using an ABI PRISM 7500 sequence detection system (Applied Biosystems, Foster City, CA, USA) in a 20 μl reaction volume consisting of the following reagents: 300 nM (each) target primer (HLBas and HLBr), 150 nM target probe (HLBp), and 1× TaqMan qPCR Mix (Applied Biosystems). The amplification protocol was as follows: 95 °C for 20 s followed by 40 cycles at 95 °C for 3 s and 60 °C for 30 s. All reactions were performed in triplicate and each run contained one negative (DNA from healthy plant) and one positive (DNA from *Ca*. L. asiaticus-infected plant) control. The positive control was same for all the runs, and was checked to make sure that the *C*_*t*_ remained constant. Data were analyzed using the ABI 7500 Fast Real-Time PCR System with SDS software.

After grafting 6 months, the scion survival, scion grown rate, scion infected, Las transmission, and disease index were calculated according to our previous studies^11,22^.

### Data analysis

Data analysis was conducted similarly to our previous study, with minor revisions^11^. Variance analysis was conducted to analyze the antibacterial activity and phytotoxicity of chemical compound. The data of antimicrobial compound treatments were analyzed by Duncan’s multiple range test at *P* < 0.05. In the further evaluation, the antibacterial and phytotoxicity of the chemical treatments were carried out by principal component and hierarchical cluster analyses using the SAS/STAT procedure in PRINCOMP and CLUSTER, respectively. the membership function for each index was calculated using the following equation: U(X_i_) = (X_i_ − X_min_)/(X_max_ − X_min_) (i = 1, 2, 3, …n), where X_i_ is the measured index value, and X_min_ and X_max_ are the minimum and maximum values of one given index for all tested materials, respectively. The comprehensive evaluation value of efficacy of antimicrobial compound against HLB were calculated by following equation:

$$D(X)=\,\sum _{j=1}U(Xj)\,\ast \,Wj(j=1,2,3\ldots n).$$

Furthermore, seven variables of antibacterial activities and phytotoxicity (scion survival, scion growth, infection rates; Las transmission; *Ct* values in scions and rootstocks; and disease index) were accessed at each step of the stepwise discriminant analysis process. All the data analysis was run in SAS software package (SAS V.9.1, SAS institute, NC, USA).

## Results

### Survival and growth of scions treated with antimicrobial compounds

CRE treatment displayed significant phytotoxicity to scions. Only 18.75% of the scions survived and little new flush (12.50 ± 0.0%) was produced with this treatment. However, more than 50% of the scions that were treated with the other remaining chemicals survived. Although scion growth rates with CRE, as well as AL, ABC, 20WP3, EcoClean3, SN, 80WG, SAR, EBI-601, EBI-602, and MA were all less than 20%, the scion survival rate with the latter antimicrobial compounds was 54.55–99.47%. In addition, several chemicals, including MET, BER, HYD, INH, SAP, THU, SD, and PT81, demonstrated higher scion survival and growth rates, as well as the positive control Amp (Table 2).

**Table 2:** Efficacy of chemical compounds against Las bacterium.

Chemical compounds	Scion survival (%)	Scion grown rate (%)	Scion infected (%)	Las transmission (%)	Ct value in scion	Ct value in rootstock	Disease index
AL	66.67	16.67 ± 0	33.34 ± 0	11.12 ± 0	33.87 ± 1.09	36.45 ± 0.57	16.67
Amp	97.5	48.8 ± 3.66	0 ± 0	0 ± 0	40 ± 0	40 ± 0	0
AZA	95.83	37.96 ± 4.18	81.75 ± 5.62	81.67 ± 2.36	28.11 ± 0.66	24.85 ± 0.03	37.96
CARV	63.64	22.73 ± 0	40 ± 0	23.74 ± 17.86	32.53 ± 2.51	35.25 ± 3.84	22.73
MESO	95.45	47.23 ± 3.93	72.5 ± 3.54	95 ± 7.08	28.64 ± 0.95	23.94 ± 0.77	47.23
PCY	75	41.67 ± 3.93	25.9 ± 16.42	38.89 ± 7.86	34.65 ± 1.31	33.45 ± 0.7	41.67
PDL	80	50 ± 21.22	44.51 ± 2.34	20 ± 0	33.46 ± 0.55	35.5 ± 0.85	50
PLA	70	35 ± 7.08	39.59 ± 32.41	25 ± 7.08	33.75 ± 2.91	35.76 ± 0.35	35
THU	77.27	60.99 ± 3.75	67.86 ± 15.16	70.08 ± 16.61	28.37 ± 2.23	27.18 ± 3.1	60.99
SFC	54.55	25 ± 5.9	17.15 ± 4.05	25 ± 11.79	35.22 ± 0.83	34.36 ± 0.14	25
NS	99.47	16.78 ± 13.29	61.11 ± 34.74	68.31 ± 19.94	31.98 ± 4.27	30.4 ± 2.79	48.43
SC	66.67	25 ± 11.79	16.67 ± 23.58	44.45 ± 15.72	36.08 ± 0.91	33.89 ± 2.03	25
SD	77.54	41.08 ± 9.73	42.62 ± 21.17	15 ± 5	35.68 ± 1.91	38.53 ± 0.46	13.49
SDN	65	35 ± 7.08	50 ± 0	35 ± 7.08	31.16 ± 2.51	32.56 ± 0.04	35
SN	50	38.13 ± 9.73	42.23 ± 3.15	31.25 ± 26.52	32.22 ± 1.34	33.63 ± 2.09	38.13
ABC	54.55	13.64 ± 0	100 ± 0	63.64 ± 0	23.64 ± 1.22	28.21 ± 2.03	13.64
SAR	95.07	12.54 ± 5.66	42.78 ± 15.49	46.54 ± 10.05	33.53 ± 1.51	33.6 ± 1.19	28.76
BSO	50	29.55 ± 3.22	15.48 ± 1.69	22.73 ± 6.43	35.61 ± 0.39	35.68 ± 0.36	29.55
BER	81.82	65.91 ± 9.65	53.37 ± 22.44	68.19 ± 6.43	30.06 ± 2.49	26.45 ± 0.96	65.91
MET	63.64	52.28 ± 3.22	78.41 ± 4.83	81.82 ± 0	27.33 ± 1.66	24.8 ± 1.7	52.28
NIC	50	20.46 ± 3.22	10 ± 14.15	13.64 ± 6.43	36.36 ± 0.55	36.24 ± 0.24	20.46
EBI-601	63.34	13.34 ± 2.89	66.67 ± 57.74	23.34 ± 32.15	37.18 ± 4.89	37.98 ± 1.13	10.69
EBI-602	85	10 ± 17.33	5.57 ± 9.65	40 ± 36.06	37.19 ± 0	33.77 ± 5.12	24.9
2AC	77.27	24.32 ± 15.11	52.39 ± 26.94	80.91 ± 1.29	27.51 ± 2.04	26.27 ± 0.5	24.32
HYD	83.33	56.82 ± 9.65	67.86 ± 25.26	52.66 ± 15.54	29.8 ± 2.41	28.96 ± 0.84	56.82
INH	90.91	70.84 ± 17.68	43.19 ± 9.65	70.84 ± 5.9	32.14 ± 2.29	26.93 ± 1.29	70.84
QUI	66.67	34.17 ± 1.18	46.43 ± 5.06	23.34 ± 9.43	31.66 ± 1.76	34.33 ± 1.1	34.17
CRE	18.75	12.5 ± 0	100 ± 0	43.75 ± 8.84	26.95 ± 1.2	30.67 ± 0.79	12.5
FA	95.45	36.6 ± 26.04	86.37 ± 19.29	95.46 ± 6.43	26.17 ± 1.27	24.29 ± 0.16	36.6
ZINEB	90	42.5 ± 17.68	74.25 ± 10.72	95 ± 7.08	28.14 ± 0.52	27.46 ± 1.78	42.5
80WG	75.56	6.27 ± 2.45	50 ± 50	65.19 ± 8.34	36.61 ± 3.39	32.91 ± 0.79	17.77
BITC	87.5	28.34 ± 16.5	31.25 ± 8.84	48.34 ± 25.93	33.55 ± 1.55	30.91 ± 5.59	28.34
QUAD	95.45	40.91 ± 19.29	91.67 ± 11.79	86.37 ± 6.43	25.41 ± 3.24	25.32 ± 2.57	40.91
SAP	95	62.5 ± 3.54	71.48 ± 18.59	100 ± 0	26.91 ± 0.31	23.27 ± 0.91	62.5
20WP	86.67	9.41 ± 2.78	81.67 ± 31.76	60.19 ± 15.3	29.93 ± 1.9	31.56 ± 2.82	43.19
EcoClean	75.56	10.42 ± 12.68	56.67 ± 40.42	59.03 ± 6.75	30.74 ± 6.96	30.54 ± 1.93	43.46
FT33	83.34	20 ± 8.67	40 ± 34.65	70.37 ± 23.17	33.99 ± 3.05	29.87 ± 3.56	45.04
MA	96.67	11.67 ± 2.89	50 ± 50	66.67 ± 30.56	30.67 ± 6.93	30.65 ± 3.5	44.15
Proud	26.78	26.78 ± 7.39	56.59 ± 26.98	62.6 ± 38.23	31.14 ± 4.33	30.27 ± 5.75	50.56
PT81	90	83.34 ± 2.89	66.67 ± 57.74	90 ± 32.15	31.07 ± 5.84	23.59 ± 2.31	72.75
DMSO	87.6	31.6 ± 6.22	75 ± 0	100 ± 0	32.97 ± 0	24.83 ± 0	56.25
CK-1	90.91	30.69 ± 8.04	55 ± 7.08	95 ± 7.08	26.9 ± 2.18	26.2 ± 2.5	72.8
CK-2	91.67	50 ± 0	55 ± 7.08	95 ± 7.08	24.99 ± 0.24	24.94 ± 0.55	80.21

### Effect of antimicrobial compounds against Las bacterium

Variance analysis indicated that the chemicals had significant effects on Las titers in scions (*P* = 0.0001) and rootstocks (*P* = 0.0001), as well as the percentage of infected scions (*P* = 0.0032) and Las transmission (*P* = 0.0001), in the fixed model. Plants grafted with Las-infected scions soaked in antimicrobial compounds Amp, EBI-601, and NIC showed a significant reduction in Las in both scions and rootstocks, resulting in a *C*_*t*_ > 36.0 (Table 2). However, the scion infection rate (10–66.67%), Las transmission rate (13.64–23.24%), and DI (10.69–20.46) of EBI-601 and NIC were much higher than those of Amp (Table 2). Las-infected scions treated with 80WG, EBI-602, and SC displayed a marked reduction in Las (*C*_*t*_ = 36.08–37.19), and the scion infection rate, Las transmission rate, and DI were 5.57–50%, 40–65.19%, and 17.77–25%, respectively. Las titers in rootstocks grated by Las-infected scions soaked in SD and AL were also remarkably reduced with 11–15% Las transmission and 13.49–16.67% DI (Table 2). Some antimicrobial compounds, including 2AC, MET, AZA, FA, INH, MESO, QUAD, SAP, THU, ZINEB, PT81, and FT33, were not effective in suppressing Las, leaving more than 70.0% of the rootstocks infected (Table 2) and 24.32 to 72.75% DI. None of the negative controls (0.1% DMSO, 0.1% CK-1 and CK-2) had a significant effect on Las titers in the incubated rootstocks (*C*_*t*_ = 24.83–26.20) or scions (*C*_*t*_ = 24.99–32.7), and the DI of these solvents ranged from 56.25 to 80.21%.

### Principal component, hierarchical cluster, membership function and stepwise discriminant analyses

Principal component analysis was used for the data obtained from the 39 tested compounds and 4 controls (CK) after standardization as described in the Methods. The first principal component accounted for 58.30% of the total variance in the data set, while the second principal component explained 19.18% (Table 3). The contribution of each variable, their relationships, and the resulting principal components are shown in (Table 3). The scion infection rate, Las transmission rate, and disease index contributed primarily to the first principal component, as did the percentage of *C*_*t*_ values in scions and rootstocks, but with opposite values to *C*_*t*_ value. Scion growth and survival contributed primarily to the second principal component, as did the scion infection and Las transmission rates, but with opposite values to the scion infection and Las transmission.

**Table 3:** Result of principal component analysis.

Principal component	Scion survival(%)	Scion grown rate (%)	Scion infected(%)	Las transmission (%)	Ct value in scion	Ct value in rootstock	Disease index	Accumulative contribution rate(%)
1	0.2239	0.255	0.3586	0.4589	−0.4122	−0.477	0.385	58.3005
2	0.4852	0.5203	−0.4877	−0.0532	0.3648	0.0498	0.3432	77.4763

**Figure 1:**
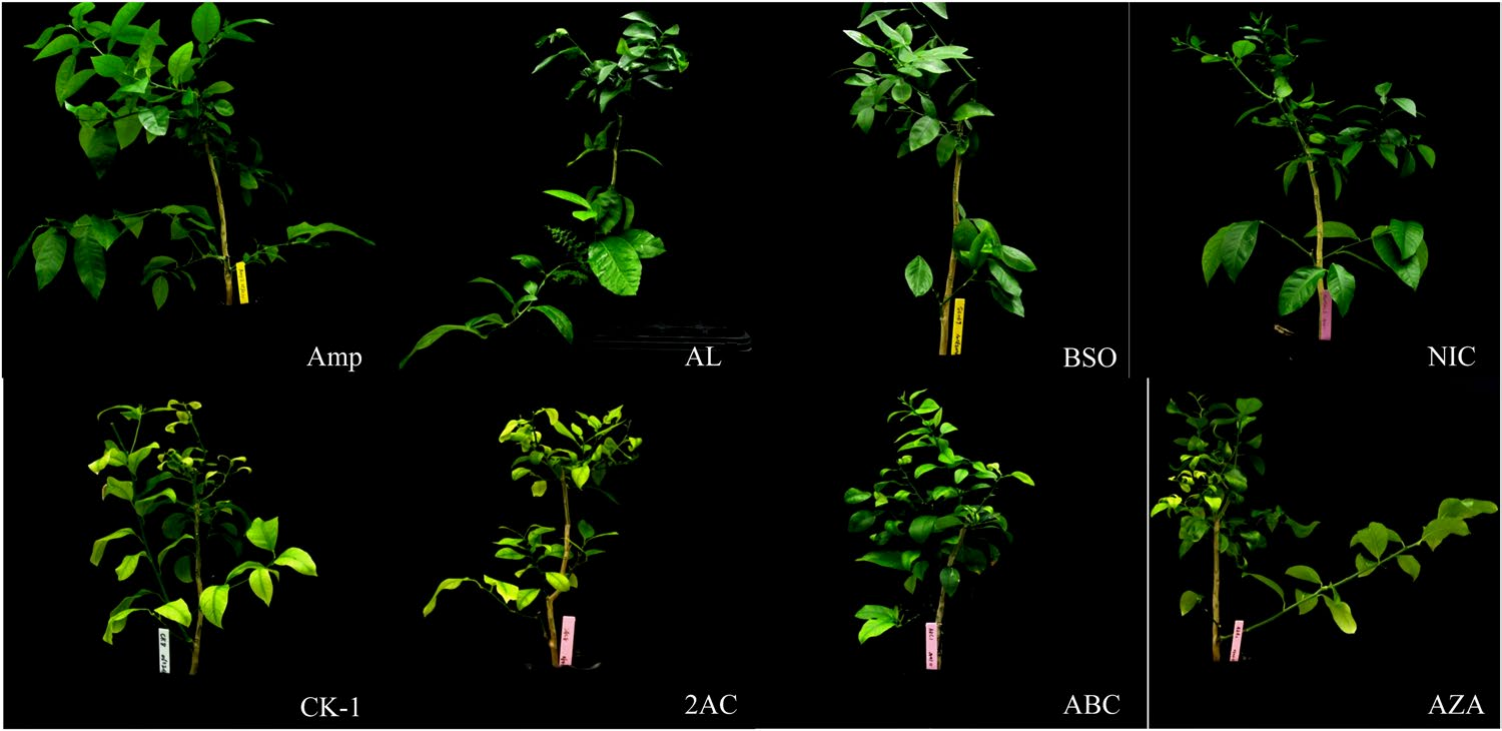
Huanglongbing (HLB)-affected grapefruit (‘Duncan’) plants with grafted-inoculation of Las-infected lemon scions treated with various chemical compounds. Amp: Ampicillin (positive control); AL: Aluminum hydroxide (effective); BSO: DL-buthionine-sulfoximine (effective); NIC: Nicotine (effective); CK-1: Water (negative control); 2AC; 2-amino-5-chlorobenzoxazole (ineffective); ABC: DL-2-aminobutyric acid (ineffective); AZA: Azadirachtin (ineffective).

In the principal component, hierarchical cluster, and membership function analyse, antimicrobial compounds were classified by scion infection rate, Las transmission rate, *C*_*t*_ values of scions and rootstocks, and DI without considering information regarding the antimicrobial compound class. The result indicated that 39 antimicrobials were divided into three groups: “effective” (Group I), “partly effective” (Group II), and “ineffective” (Group III). Group I was composed of 9 antimicrobial compounds (AL, SD, EBI-601, BSO, SFC, NIC, SC, EBI-602, and Amp) which displayed high antibacterial activity against Las, resulting in the lowest Las titers in scions and rootstocks of grafted plants (Tables 4 and 5). Group III consisted of 24 antimicrobial compounds and had the least antibacterial effect; this group had the highest scion infection rate, Las transmission rate, and Las titers in citrus grafted-scions (Tables 4 and 5). Group II compounds (BITC, CARV, QUI, PDL, PCY, PLA, SN, SDN, 80WG, and SAR) partially suppressed Las as compared to Groups I and III (Tables 4 and 5). In addition, the scions and rootstocks of plants grafted to scions treated with Group I compounds, did not displayed HLB-like symptoms (Fig. 1), while those grafted to negative control or Group III solvent-soaked Las-positive scions had typical HLB symptoms, such as yellow shoots, corky leaves in rootstocks, or blotchy mottled leaves on the scion.

**Table 4:** Membership function of principal component, comprehensive evaluation, hierarchical cluster and stepwise discriminant analyses.

Chemical compounds	U (1)	U (2)	Integrated assessment value (D)	Rank	SDA^b^	
					Group	Posteriori probability
Amp	0	1	0.192618	1	I	0.9995
NIC	0.066726681	0.63919921	0.083788765	2	I	0.9657
BSO	0.160210512	0.671083	0.034825458	3	I	0.8211
EBI-602	0.203226923	0.77348967	0.029194502	4	I	0.8606
SD	0.187632435	0.71332287	0.026797572	5	I	0.9464
SFC	0.180956997	0.64393869	0.017367815	6	I	0.8339
AL	0.157842255	0.57289262	0.017308209	7	I	0.7483
EBI-601	0.15057129	0.47288647	0.002331145	8	I	0.8606
SC	0.241922305	0.70418244	−0.006964583	9	I	0.8293
CARV	0.259164878	0.55511956	−0.045840531	10	II	0.652
PLA	0.30675161	0.69019481	−0.04787294	11	II	0.8719
PCY	0.371844055	0.80732773	−0.063680228	12	II	0.8216
PDL	0.405332041	0.819992	−0.08098059	13	II	0.9792
80WG	0.322969323	0.53854676	−0.086642728	14	II	0.8297
QUI	0.357814556	0.61367064	−0.092712284	15	II	0.9401
SN	0.353535074	0.58855754	−0.095026949	16	II	0.9404
SAR	0.38598435	0.66211184	−0.099986519	17	II	0.8726
BITC	0.418405928	0.7250177	−0.106980844	18	II	0.7348
SDN	0.424181151	0.58663404	−0.137040272	19	II	0.8992
FT33	0.516570875	0.6923799	−0.171131487	20	III	0.5476
EcoClean	0.525268333	0.51617271	−0.21019894	21	III	0.7774
MA	0.575431064	0.6320746	−0.217442923	22	III	0.5706
Proud	0.604251783	0.65615671	−0.22979285	23	III	0.8926
NS	0.613156984	0.66195796	−0.233924658	24	III	0.9142
CRE	0.433003255	0	−0.255236799	25	III	0.9876
20WP	0.595457378	0.46907026	−0.260645144	26	III	0.9735
INH	0.779211966	0.96680824	−0.273087279	27	III	0.9955
HYD	0.7120975	0.73020343	−0.279100532	28	III	0.996
2AC	0.649761097	0.47436371	−0.291635238	29	III	0.9655
BER	0.7898068	0.82848614	−0.305975805	30	III	0.9989
THU	0.805165847	0.70316684	−0.339168055	31	III	0.9995
DMSO	0.803102278	0.6469855	−0.348773206	32	III	1
ABC	0.639927983	0.09995999	−0.357955935	33	III	0.9991
ZINEB	0.810816425	0.59682085	−0.362982978	34	III	0.9987
AZA	0.824305796	0.5641215	−0.377232866	35	III	1
CK-1	0.874537774	0.67931554	−0.384654011	36	III	0.9996
PT81	0.960182813	0.9177669	−0.389208055	37	III	1
MET	0.832961706	0.52569784	−0.389736241	38	III	1
MESO	0.884010803	0.65975749	−0.394005188	39	III	1
FA	0.893996261	0.50323147	−0.430040915	40	III	1
CK-2	0.980486116	0.75240821	−0.43302704	41	III	0.9999
QUAD	0.90139187	0.50998677	−0.433099117	42	III	1
SAP	1	0.73985166	−0.446948253	43	III	1

^b^SDA: stepwise discriminant analysis: the group is classified by SDA.

**Table 5:** Chemical compound classification of antibacterial activity against Las bacterium.

Variables	Group I	Group II	Group III
Scion survival (%)	67.92 ± 16.22a^b^	72.85 ± 12.85a	80.98 ± 20.92a
Scion grown rate (%)	25.55 ± 12.72a	30.39 ± 13.31a	40.62 ± 22.58a
Scion infected (%)	23.06 ± 21.01c	41.27 ± 7.71b	68.29 ± 16.65a
Las transmission (%)	21.7 ± 13.97c	35.73 ± 14.28b	77.19 ± 16.28a
Ct value in scion	36.36 ± 1.71a	33.31 ± 1.57b	28.9 ± 2.66c
Ct value in rootstock	36.32 ± 2.18a	33.79 ± 1.5b	27.14 ± 2.65c
Disease index	18.42 ± 9.25c	33.16 ± 9.3b	49.24 ± 17.45a
Compounds included	AL, BSO, NIC, SC, SFC, SD, EBI-601, EBI-602, Amp	BITC, CARV, QUI, PDL, PCY, PLA, SN, SDN, 80WG, SAR	2AC, MET, AZA, BER, ABC, FA, HYD, INH, CRE, MESO, QUAD, SAP, THU, ZINEB, 20WP, Proud, EcoClean, NS, PT81, FT33, MA, DMSO, CK-1, CK-2
Group Classification	Highly effective	Partly effective	Non-effective

^b^Different letter by group indicated that the significance at 0.05 level.

The results from stepwise discriminant analysis indicated the scion infection rate, *C*_*t*_ of the inoculated rootstock, and DI were selected for discriminant function based on Wilk’s lambda and the F-value (*P* = 0.00010 and *χ*^2^ = 74.942; Table 6). By using these three variables as predictors, 100% of the antimicrobial compounds were correctly classified into hierarchical cluster analysis groups from all seven variables. Also, 23 out of 39 compounds were correctly classified as having an overall a posteriori probability greater than 90.0%.

**Table 6:** Selected variable of antibacterial activity by stepwise discriminant analysis at *Chi* = 74.942 and P = 0.00010.

Variable	Wilks Lambda	F value	Selected (N/Y)
Scion survival (%)	0.9975	0.0462	N
Scion grown rate (%)	0.9029	1.9887	N
Scion infected (%)	0.6325	11.0376	Y
Las transmission (%)	0.9665	0.6417	N
Ct value in scion	0.9687	0.5978	N
Ct value in rootstock	0.7334	6.9064	Y
Disease index	0.8156	4.2948	Y

## Discussion

Citrus HLB is a devastating disease of citrus worldwide. Chemical control is considered to be an effective short-term strategy against Las bacterium. Antibiotics have been used in several agricultural practices for decades, and their use has only begun to peter out due to public concerns about emergence of antibiotic-resistant bacteria and potential side effects on humans. Thus, non-antibiotic chemical compounds that can reduce or eliminate the risk of creating antibiotic-resistant bacteria and have little to no negative effects on humans are needed to rescue the citrus industry. In the present study, 39 antimicrobial compounds (including natural product, antimicrobial metal, and commercial product) that are already approved and being applied to commercial agricultural products were evaluated for their efficacy against Las and phytotoxicity to citrus trees. Principal component, hierarchical cluster and membership function analyses clustered these compounds into three groups based on their anti-Las activity and citrus phytotoxicity. Group I compounds (AL, BSO, NIC, SC, SFC, SD, EBI-601, and EBI-602) were highly effective, along with the positive control (Amp), yielding the lowest Las titers in inoculated plants. Group II compounds (BITC, CARV, QUI, PDL, PCY, PLA, SN, SDN, 80WG, and SAR) were partly effective; and Group III chemicals (2AC, MET, AZA, BER, ABC, FA, HYD, INH, CRE, MESO, QUAD, SAP, THU, ZINEB, 20WP3, Proud, EcoClean, NS, PT81, FT33, and MA), along with negative controls (DMSO, CK-1, and CK-2), were relatively ineffective and showed the highest Las titers.

With antibiotic-resistant bacteria posing a significant public health challenge, interest in understanding the antimicrobial properties associated with certain metals, such as silver and aluminum, is increasing. In ionic or nanoparticle forms, silver displays strong activity against microorganisms and has been used as a medicinal and antibacterial agent since the nineteenth century^23,24^. Silver can influence a broad range of biological processes in microorganisms, such as cell membrane structure and function^25–27^. The expression of proteins involved in ATP production is also inhibited by silver^28^. In the present study, SC and SD were highly effective at suppressing Las bacterium and showed little phytotoxicity to citrus (Tables 2 and 5). SN and SDN (Silver, nanoparticle from Fisher Scientific) belonged to Group II and were partly effective against Las bacteria, while nanosilver from Allostat was not effective (Tables 2 and 5). Although the various antibacterial activities of silver compounds obtained from different companies likely result from their various chemical and physical characteristics, the mechanism(s) by which they exert their effect on Las bacterium is unknown. Both SC and SD are colloidal forms of silver. Generally, SC is a suspension of submicroscopic silver nanoparticles in water, with diameters ranging from 10 to 100 nm^29^. Furthermore, SC reportedly has a broad effect against a wide spectrum of bacteria, including antibiotic-resistant forms^30,31^. However, the safety of SC in humans and the environment is still a public concern. Bactericidal doses of silver ions range from 1000 to 10,000 mg/L in water^32^; at higher doses, silver can be toxic to mammals^33,34^ and freshwater and marine organisms^35,36^. Silver concentrations of less than 200 mg/L have no harmful effects on humans^37^. The present study used SC and SD concentrations less than 100 mg/L (Table 1). Therefore, their use against HLB in citrus can be considered safe for humans. In the future, application of SC and SD on HLB-affected citrus trees in greenhouses and the field will be conducted, as well as a more intensive evaluation of their safety in humans and the environment.

Aluminum (AL) was also effective against Las bacterium and showed little phytotoxicity towards citrus. The *C*_*t*_ of inoculated rootstocks and infected scions treated with AL were 36.45 ± 0.57 and 33.87 ± 1.09, respectively (Table 2), and the DI was only 16.17. However, the scion growth rate of HLB-infected scions soaked in AL was less than 20% (Table 2). The antimicrobial activity of aluminum due to the release of metal ions has been addressed in a few previous studies. Positively charged aluminum ions attach to the surface of bacteria due to their negative surface charge at physiological pH^38,39^. Therefore, aluminum plays an important role in bacterial toxicity. Although AL shows bactericidal activity against Las, its toxicity to citrus plants, humans, and the environment must be evaluated further.

NIC, 3-(1-methyl-2-pyrrolidinyl) pyridine, is a colorless to light-pale yellow, hygroscopic, yet oily liquid naturally present in the leaves of *Nicotiana tabacum*. It is considered to be a highly toxic chemical, which was belonged to the tobacco alkaloids^40^. Several studies have demonstrated that NIC can suppress growth of microorganisms, including bacteria^41–43^. In our study, NIC was found to effectively suppress Las titers in inoculated rootstocks and scions, with a DI of only 20.46 (Table 2). Previously, 45 °C thermotherapy combined with NIC applied to HLB-affected citrus by bark painting was shown to have a much higher therapeutic efficiency against Las bacterium than this combination treatment at 40 or 42 °C. The increase in therapeutic effect was attributed to an increased ability to uptake NIC through the bark at higher temperatures^22^. Therefore, how different antimicrobials, especially NIC, are delivered into the citrus phloem will be investigated in future studies.

BSO has been shown to reduce glutathione levels and is being investigated as an adjunct to chemical control for the treatment of cancer^44^. Glutathione has a broad range of biochemical functions^45,46^. In particular, it is a major cellular antioxidant and determinant of redox state^47,48^. Glutathione can prevent damage to plant caused by reactive oxygen species (ROS). Therefore, BSO is a glutathione-depleting agent that can enhance production of ROS that have potent antimicrobial activity agaist bacteria. Our results showed that Las titers were reduced in inoculated rootstocks and scions soaked in BSO, and BSO had phytotoxicity (Table 2). This may be attributed to production of reactive oxygen species effective against Las bacterium and related damage to citrus tree cells. Furthermore, this probable bactericidal mechanism of BSO is not likely to result in the emergence of antibiotic resistant bacteria. Therefore, BSO may have a great value in the rescue and maintenance of citrus crops.

SFC is an antimicrobial lipopeptide family member produced by *Bacillus subtilis* that displays antibacterial, antiviral, antitumor, and hemolytic action^49^. Given its biological origin, SFC is generally considered to be of lower risk to humans and the environment than antibiotics. SFC’s ability to penetrate the cell membrane of all types of bacteria^50^ gives it very significant antibacterial activity. In previous studies, SFC from *Bacillus subtilis* was shown to reduce infections caused by *Pseudomonas syringae* on *Arabidopsis* plants^51^. SFC can also interact with plant cells as a bacterial deterrent by stimulating induction of systemic immune resistance^52,53^. Our current results demonstrated that SFC from *Bacillus subtilis* displayed effective antibacterial activity against Las bacterium and low phytotoxicity in citrus plants (Table 2), likely resulting from its induction of systemic resistance. Therefore, the eco-friendly antimicrobial SFC is a potential candidate for control of citrus HLB in the field.

EBI-601 and EBI-602 also belonged to Group I, being highly effective against Las bacterium (Tables 2 and 5). These chemicals are both degradation products of tetracycline. Previous studies have demonstrated that although tetracycline can suppress Las bacterium, it shows serious citrus phytotoxicity^11,17,20^. Our research demonstrated that EBI-601 and EBI-602 could not only suppress Las titers in inoculated rootstocks and scions, but also had low phytotoxicity to citrus plants (Table 2). As degradation products of tetracycline, the physical and chemical characteristics of EBI-601 and EBI-602 may be different from that of tetracycline. Thus, the mechanism of their effect on Las and citrus plants is unknown.

Two other chemical compounds that were screened included the essential oils CARV and PCY. Essential oils are extracted from aromatic plants. Therefore, essential oil is one of most important natural compounds and have antioxidant, antiradical, and antimicrobial properties. Currently, they have been largely utilized in food, cosmetics, and pharmaceuticals^54,55^. Several essential oils, including CARV and PCY, displayed strong antibacterial activity^56,57^. This has been attributed to their ability to permeabilize and depolarize cytoplasmic membranes^58^. Several previous studies have demonstrated that essential oils also have insecticidal properties^59,60^. However, the effect of essential oils, especially CARV and PCY, on the citrus psyllid, which is a vector for transmitting Las bacterium, is still unknown. Our current results indicated that CARV and PCY were partially effective against Las bacterium. Furthermore, CARV and PCY can be prepared as nanoemulsions, enhancing their delivery efficiency and antibacterial activities^61,62^. Therefore, CARV and PCY are ideal candidates for combating HLB due to their eco-friendly, antibacterial and insecticidal properties, nanoemulsion characteristics, and ability to reduce Las titers.

Public concerns regarding emergence of antibiotic resistant bacteria due to the overuse of antibiotics on plants in the open environment and over large expanses of land has limited their applications in agricultural practices. In the present study, several effective and partially effective non-antibiotic antimicrobial compounds against Las bacterium were identified. These antimicrobials include metals and natural products that may reduce the risks associated with emergence of antibiotic resistance. However, the anti-Las activities of Groups I and/or II are still lower than that of Amp (positive control). These antimicrobials have anti-Las activity, low citrus phytotoxicity, and are generally considered safe for humans and the environment. It is possible that using a nano-delivery system or combining their application with thermotherapy would enhance the bactericidal activity of these compounds. The present research identified several highly and partially effective antimicrobials that may be effective for control of citrus HLB in the field by foliar spray or trunk injection. Future studies must assess potential risks these antimicrobials may pose to humans and the environment.

## References

[1] Bove JM (2006). Huanglongbing: A destructive, newly-emerging, century-old disease of citrus. J. Plant. Pathol..

[2] McClean A, Oberholzer P (1965). Citrus psylla, a vector of the greening disease of sweet orange. South. Afri. J. Agri. Sci..

[3] Chen Q (1943). A report of a study on yellow shoot of citrus in Chaoshan. New. Agric. Q. Bull..

[4] Neupane, D., Moss, C. B. & Bruggen, V. Estimating citrus production loss due to citrus huanglongbing in Florida. Southern Agricultural Economics Association (SAEA) Annual Meeting. 6–9 (San Antonio, Texas, February, 6-9, 2016).

[5] United States Department of Agriculture, Washington D.C. Published online, http://www.nass.usda.gov/Statistics_by_State/Florida/Publications/Citrus/cit/2015-16/cit1015.pdf (2015).

[6] Bove JM, Ayres AJ (2007). Etiology of three recent diseases of citrus in Sao Paulo State: sudden death, variegated chlorosis and huanglongbing. IUBMB. life..

[7] Jagoueix S, Bove JM, Garnier M (1994). The phloem-limited bacterium of greening disease of citrus is a member of the *alpha* subdivision of the *Proteobacteria*. Int. J. Syst. Bacteriol..

[8] Gottwald TR (2007). Citrus canker and citrus huanglongbing, two exotic bacterial diseases threatening the citrus industries of the Western Hemisphere. Outlooks on Pest Management..

[9] Davey MR, Anthony P, Power JB, Lowe KC (2005). Plant protoplasts: status and biotechnological perspectives. Biotechnol. Adv..

[10] Zhang M, Powell CA, Guo Y, Doud MS, Duan Y (2012). A graft-based chemotherapy method for screening effective molecules and rescuing huanglongbing-affected citrus plants. Phytopathology..

[11] Zhang M (2014). Effective Antibiotics against ‘*Candidatus* Liberibacter asiaticus’ in HLB-Affected Citrus Plants Identified via the Graft-Based Evaluation. PloS one..

[12] Yang C, Powell CA, Duan Y, Shatters R, Zhang M (2015). Antimicrobial Nanoemulsion Formulation with Improved Penetration of Foliar Spray through Citrus Leaf Cuticles to Control Citrus Huanglongbing. PloS one..

[13] Yang C, Powell C, Duan Y, Zhang M (2016). Characterization and Antibacterial Activity of Oil-In-Water Nano-Emulsion Formulation Against *Candidatus* Liberibacter asiaticus. Plant. Dis..

[14] Hu J, Jiang J, Wang N (2017). Control of Citrus Huanglongbing via Trunk Injection of Plant Defense Activators and Antibiotics. Phytopathology..

[15] Hu J, Wang N (2016). Evaluation of the spatiotemporal dynamics of oxytetracycline and its control effect against citrus Huanglongbing via trunk injection. Phytopathology..

[16] Li J, Trivedi P, Wang N (2015). Field evaluation of plant defense inducers for the control of citrus huanglongbing. Phytopathology..

[17] Schwarz R, Van Vuuren S (1971). Decrease in fruit greening of sweet orange by trunk injection of tetracyclines. Plant. Dis. Rep..

[18] Zhao, X. Y. Citrus yellow shoot disease (Huanglongbing) in China-a review. *International Society of Citriculture*. 466–469 (Tokyo, Japan, November 9–12, 1981).

[19] Aubert, B. & Bové, J. Effect of penicillin or tetracycline injections of citrus trees affected by greening disease under field conditions in Reunion Island. In Proc. 8th Conference of the International Organization ofCitrus Virologists (eds Calavan, E. C., Garnsey, S. M. & Timmer, L. W.) 103–108 (IOCV, Riverside, CA, 1980)

[20] Martinez A, Nora D, Armedilla A (1970). Suppression of symptoms of citrus greening disease in the Philippines by treatment with tetracycline antibiotics. Plant. Dis. Rep..

[21] Li W, Hartung JS, Levy L (2006). Quantitative real-time PCR for detection and identification of *Candidatus* Liberibacter species associated with citrus huanglongbing. J. Microbiol. Meth..

[22] Yang C (2016). Mitigating citrus huanglongbing via effective application of antimicrobial compounds and thermotherapy. Crop. Prot..

[23] Oloffs A (1994). Biocompatibility of silver-coated polyurethane catheters and silvercoated Dacron® material. Biomaterials..

[24] Tokumaru T, Shimizu Y, Fox C (1974). Antiviral activities of silver sulfadiazine in ocular infection. Res. Commun. Chem. Pathol. Pharm..

[25] McDonnell G, Russell AD (1999). Antiseptics and disinfectants: activity, action, and resistance. Clin. Microbiol. Rev..

[26] Pal S, Tak YK, Song JM (2007). Does the antibacterial activity of silver nanoparticles depend on the shape of the nanoparticle? A study of the gram-negative bacterium *Escherichia coli*. Appl. Environ. Microbiol..

[27] Sondi I, Salopek-Sondi B (2004). Silver nanoparticles as antimicrobial agent: a case study on E. coli as a model for Gram-negative bacteria. J. Colloid. Interf. Sci..

[28] Yamanaka M, Hara K, Kudo J (2005). Bactericidal actions of a silver ion solution on Escherichia coli, studied by energy-filtering transmission electron microscopy and proteomic analysis. Appl. Environ. Microbiol..

[29] Zhang X-F, Liu Z-G, Shen W, Gurunathan S (2016). Silver Nanoparticles: Synthesis, Characterization, Properties, Applications, and Therapeutic Approaches. Int. J. Mol. Sci..

[30] Wiemken TL (2015). Efficacy of a novel skin antiseptic against carbapenem-resistant Enterobacteriaceae. Am. J. Infect. control..

[31] Huang L, Dai T, Xuan Y, Tegos GP, Hamblin MR (2011). Synergistic combination of chitosan acetate with nanoparticle silver as a topical antimicrobial: efficacy against bacterial burn infections. Antimicrob. Agents. Chem..

[32] Liu Z (1994). Controlled evaluation of copper-silver ionization in eradicating *Legionella pneumophila* from a hospital water distribution system. J. Infect. Dis..

[33] Conrad AH (1999). Ag+ alters cell growth, neurite extension, cardiomyocyte beating, and fertilized egg constriction. Aviat. Space. Envir. Med..

[34] Hirasawa F (1997). The effect of silver administration on the biosynthesis and the molecular properties of rat ceruloplasmin. Bioch. Biophy. Acta..

[35] Bianchini A, Grosell M, Gregory SM, Wood CM (2002). Acute silver toxicity in aquatic animals is a function of sodium uptake rate. Environ. Sci. Technol..

[36] Morgan TP, Guadagnolo CM, Grosell M, Wood CM (2005). Effects of water hardness on toxicological responses to chronic waterborne silver exposure in early life stages of rainbow trout (*Oncorhynchus mykiss*). Environ. Sci. Technol..

[37] Berger T, Spadaro J, Chapin S, Becker R (1976). Electrically generated silver ions: quantitative effects on bacterial and mammalian cells. Antimicrob. Agents. Chem..

[38] Jiang W, Mashayekhi H, Xing B (2009). Bacterial toxicity comparison between nano-and micro-scaled oxide particles. Environ. Pollut..

[39] Mukherjee A (2011). Mohammed Sadiq, I., Prathna, T. & Chandrasekaran, N. Antimicrobial activity of aluminium oxide nanoparticles for potential clinical applications. Science against microbial pathogens: communicating current research and technological advances..

[40] Willits C, Swain ML, Connelly J, Brice B (1950). Spectrophotometric determination of nicotine. Anal. Chem..

[41] Pavia C, Pierre A, Nowakowski J (2000). Antimicrobial activity of nicotine against a spectrum of bacterial and fungal pathogens. J. Med. Microbiol..

[42] Roberts D, Cole P (1979). Effect of tobacco and nicotine on growth of Haemophilus influenzae *in vitro*. J. Clin. Pathol..

[43] Bardell D (1981). Viability of six species of normal oropharyngeal bacteria after exposure to cigarette smoke *in vitro*. Microbios..

[44] Defty C, Marsden J (2012). Melphalan in regional chemotherapy for locally recurrent metastatic melanoma. Curr. Top. Med. Chem..

[45] Ogawa KI (2005). Glutathione-associated regulation of plant growth and stress responses. Antioxidants & redox signaling..

[46] Wójcik M, Tukiendorf A (2011). Glutathione in adaptation of *Arabidopsis thaliana* to cadmium stress. Biol.Plantarum..

[47] Noctor G, Gomez L, Vanacker H, Foyer CH (2002). Interactions between biosynthesis, compartmentation and transport in the control of glutathione homeostasis and signalling. J. Exp. Bot..

[48] Meyer AJ (2008). The integration of glutathione homeostasis and redox signaling. J. Plant. Physiol..

[49] Seydlová G, Svobodová J (2008). Review of surfactin chemical properties and the potential biomedical applications. Open Medicine..

[50] Buchanan E, Gibbons N, Stewart W (1994). Bergeys. Manual of Determinative Bacteriology (Edited by R. Sol Energy)..

[51] Bais HP, Fall R, Vivanco JM (2004). Biocontrol of *Bacillus subtilis* against infection of Arabidopsis roots by Pseudomonas syringae is facilitated by biofilm formation and surfactin production. Plant. Physiol..

[52] Van Loon, L. & Bakker, P. In *PGPR: Biocontrol and biofertilization*. 39–66 (Springer, 2005).

[53] Ongena M (2007). Surfactin and fengycin lipopeptides of *Bacillus subtilis* as elicitors of induced systemic resistance in plants. Environ. Microbiol..

[54] Burt S (2004). Essential oils: their antibacterial properties and potential applications in foods—a review. Int. J. Food. Microbiol..

[55] Bakkali F, Averbeck S, Averbeck D, Idaomar M (2008). Biological effects of essential oils–a review. Food. Chem. Toxicol..

[56] Kim J, Marshall M, Cornell J, Preston JFIII, Wei C (1995). Antibacterial activity of carvacrol, citral, and geraniol against *Salmonella typhimurium* in culture medium and on fish cubes. J. Food. Sci..

[57] Sartorelli P, Marquioreto AD, Amaral‐Baroli A, Lima MEL, Moreno PRH (2007). Chemical composition and antimicrobial activity of the essential oils from two species of Eucalyptus. Phytother. Res..

[58] Xu J, Zhou F, Ji BP, Pei RS, Xu N (2008). The antibacterial mechanism of carvacrol and thymol against *Escherichia coli*. Lett. Appl. Microbiol..

[59] Konstantopoulou I, Vassilopoulou L, Mavragani-Tsipidou P, Scouras Z (1992). Insecticidal effects of essential oils. A study of the effects of essential oils extracted from eleven Greek aromatic plants on *Drosophila auraria*. Experientia..

[60] Karpouhtsis I (1998). Insecticidal and genotoxic activities of oregano essential oils. J. Agr. Food. Chem..

[61] Chang Y, McLandsborough L, McClements DJ (2013). Physicochemical properties and antimicrobial efficacy of carvacrol nanoemulsions formed by spontaneous emulsification. J. Agr. Food. Chem..

[62] Donsì F, Annunziata M, Sessa M, Ferrari G (2011). Nanoencapsulation of essential oils to enhance their antimicrobial activity in foods. Lwt-Food. Sci. Technol..

[63] Spratt BG, Cromie KD (1988). Penicillin-binding proteins of gram-negative bacteria. Clin. Infect. Dis..

[64] Jerobin J (2015). Antibacterial activity of neem nanoemulsion and its toxicity assessment on human lymphocytes *in vitro*. Int. J. Nanomed..

[65] Piao J, Kawahara-Aoyama Y, Inoue T, Adachi S (2006). Bacteriostatic activities of monoacyl sugar alcohols against thermophilic sporeformers. Biosci. biotechnol. biochem..

[66] Hyldgaard M (2014). The antimicrobial mechanism of action of epsilon-poly-l-lysine. Appl. Environ. Microbiol..

[67] Swamy MK, Akhtar MS, Sinniah UR (2016). Antimicrobial properties of plant essential oils against human pathogens and their mode of action: an updated review. J. Evidence-Based. Complementary. Altern. Med..

[68] Cohen Y (1994). 3-Aminobutyric acid induces systemic resistance against *Peronospore tabacina*. Physiol. Mol. Plant. P..

[69] Yin J, Ye J, Jia W (2012). Effects and mechanisms of berberine in diabetes treatment. Acta. Pharm. Sin. B..

[70] Pilger C, Nethercott JR, Weksberg F (1986). Allergic contact dermatitis due to a biocide containing 5-chloro-2-methyl-4-isothiazohn-3-one. Contact dermatitis..

[71] Chopra I, Roberts M (2001). Tetracycline antibiotics: mode of action, applications, molecular biology, and epidemiology of bacterial resistance. Microbiol. Mol. Biol. Rev..

[72] Sun Q-Y (2007). Synthesis of novel triazole derivatives as inhibitors of cytochrome P450 14α-demethylase (CYP51). Eur J Med Chem.

[73] Shao J, Zhou B, Chu B, Yen Y (2006). Ribonucleotide reductase inhibitors and future drug design. Eur. J. Med. Chem..

[74] Youatt J (1969). A review of the action of isoniazid. Am. Rev. Respir. Dis..

[75] Keshmiri-Neghab H, Goliaei B (2014). Therapeutic potential of gossypol: an overview. Pharm. Biol..

[76] Nychas, G. Natural antimicrobials from plants. *New methods of food preservation* (ed. Gould, G. W.) 58–89 (Springer, 1995).

[77] Ricke S (2003). Perspectives on the use of organic acids and short chain fatty acids as antimicrobials. Poultry. Sci..

[78] Mulkey, M. The grouping of a series of dithiocarbamate pesticides based on a common mechanism of toxicity. US Environmental Protection Agency, Office of Pesticide Programs, Office of Prevention, Pesticides and Toxic Substances, Washington, DC (SAP Report, 2001).

[79] Guest D, Grant B (1991). The complex action of phosphonates as antifungal agents. Biol Rev..

[80] Molina-Vargas LF (2013). Mechanism of action of isothiocyanates. A review. Agronomía Colombiana..

[81] Bossche HV (1985). Biochemical targets for antifungal azole derivatives: hypothesis on the mode of action. Curr. Topics. Medl. Mycol..

[82] Segal R, Shatkovsky P, Milo-Goldzweig I (1974). On the mechanism of saponin hemolysis—I: hydrolysis of the glycosidic bond. Biochem. Pharm..

